# Temporal fractal analysis of the rs-BOLD signal identifies brain abnormalities in autism spectrum disorder

**DOI:** 10.1371/journal.pone.0190081

**Published:** 2017-12-22

**Authors:** Olga Dona, Geoffrey B. Hall, Michael D. Noseworthy

**Affiliations:** 1 McMaster School of Biomedical Engineering, McMaster University, Hamilton, Ontario, Canada; 2 Imaging Research Centre, St. Joseph’s Healthcare, Hamilton, Ontario, Canada; 3 Department of Electrical and Computer Engineering, McMaster University, Hamilton, Ontario, Canada; 4 Department of Radiology, McMaster University, Hamilton, Ontario, Canada; 5 Department of Psychology, Neuroscience & Behaviour, McMaster University, Hamilton, Ontario, Canada; University of Texas at Austin, UNITED STATES

## Abstract

**Background:**

Brain connectivity in autism spectrum disorders (ASD) has proven difficult to characterize due to the heterogeneous nature of the spectrum. Connectivity in the brain occurs in a complex, multilevel and multi-temporal manner, driving the fluctuations observed in local oxygen demand. These fluctuations can be characterized as fractals, as they auto-correlate at different time scales. In this study, we propose a model-free complexity analysis based on the fractal dimension of the rs-BOLD signal, acquired with magnetic resonance imaging. The fractal dimension can be interpreted as measure of signal complexity and connectivity. Previous studies have suggested that reduction in signal complexity can be associated with disease. Therefore, we hypothesized that a detectable difference in rs-BOLD signal complexity could be observed between ASD patients and Controls.

**Methods and findings:**

Anatomical and functional data from fifty-five subjects with ASD (12.7 ± 2.4 y/o) and 55 age-matched (14.1 ± 3.1 y/o) healthy controls were accessed through the NITRC database and the ABIDE project. Subjects were scanned using a 3T GE Signa MRI and a 32-channel RF-coil. Axial FSPGR-3D images were used to prescribe rs-BOLD (TE/TR = 30/2000ms) where 300 time points were acquired. Motion correction was performed on the functional data and anatomical and functional images were aligned and spatially warped to the N27 standard brain atlas. Fractal analysis, performed on a grey matter mask, was done by estimating the Hurst exponent in the frequency domain using a power spectral density approach and refining the estimation in the time domain with de-trended fluctuation analysis and signal summation conversion methods. Voxel-wise fractal dimension (FD) was calculated for every subject in the control group and in the ASD group to create ROI-based Z-scores for the ASD patients. Voxel-wise validation of FD normality across controls was confirmed, and non-Gaussian voxels were eliminated from subsequent analysis. To maintain a 95% confidence level, only regions where Z-score values were at least 2 standard deviations away from the mean (i.e. where |*Z*| > 2.0) were included in the analysis. We found that the main regions, where signal complexity significantly decreased among ASD patients, were the amygdala (p = 0.001), the vermis (p = 0.02), the basal ganglia (p = 0.01) and the hippocampus (p = 0.02). No regions reported significant increase in signal complexity in this study. Our findings were correlated with ADIR and ADOS assessment tools, reporting the highest correlation with the ADOS metrics.

**Conclusions:**

Brain connectivity is best modeled as a complex system. Therefore, a measure of complexity as the fractal dimension of fluctuations in brain oxygen demand and utilization could provide important information about connectivity issues in ASD. Moreover, this technique can be used in the characterization of a single subject, with respect to controls, without the need for group analysis. Our novel approach provides an ideal avenue for personalized diagnostics, thus providing unique patient specific assessment that could help in individualizing treatments.

## Introduction

Clinical and neurobiological heterogeneity in autism spectrum disorder (ASD) is a marked obstacle in trying to understand the underlining mechanisms of the disorder and a barrier in increasing treatment efficacy [1]. Early intervention with behavioral therapies has consistently reported to improve the quality of life of ASD patients [2]; therefore, development of methods for early diagnosis that focus on patient specific abnormalities has been highly prioritized around the globe. Neuroimaging research has explored anatomical and functional disturbances in ASD with the objective of elucidating the pathophysiology of the disorder. However, giving the heterogeneous nature of ASD, contrasting brain structures and brain networks have been identified as atypical by different studies.

In terms of anatomic abnormalities, recent MRI studies have found overgrowth of the brain in early stages of the disorder and abnormal decline during later stages, specifically in adolescence [3]. The decline in adolescence is characterized by cortical atrophy [4], frontal cortex volume reduction [5] and atrophy of the amygdala [6]. Additionally, diffusion tensor imaging (DTI) studies in ASD have shown reduced white matter integrity in corpus callosum [7], anterior cingulate gyri, bilateral superior temporal sulcus and temporal lobes approaching the amygdala [8]. The abnormalities detected in these anatomical regions, combined with reduced structural connectivity, suggested changes in functional connectivity that could be of interest for diagnosis and assessment of the disorder.

Functional connectivity has been widely studied using either task-based or resting estate functional MRI techniques. However, reported results have been inconsistent or inconclusive. For instance, while some task based-fMRI studies have shown increased connectivity on ASD patients respect to controls [9–11] others have reported decreased or weaker connectivity [12–16]. Furthermore, rs-fMRI studies have reported results that range from broad reduced connectivity in the default mode network (DMN) [17] to reduced connectivity between the superior frontal gyrus and the posterior cingulate cortex (PCC) and stronger connectivity between right parahippocampal gyrus and the PCC [18].

One of the flaws of the current methodologies to assess brain connectivity in ASD patients is that they heavily rely on group analysis. Large datasets of rs-fMRI are assessed using probabilistic independent component analysis (PICA) to find temporal synchrony among regions of the brain [19, 20]. This methodology determines brain networks and its correlation coefficients, where the latter represents the connectivity strength within each network. This approach is generally unsuitable when drawing conclusions on such heterogeneous disorders and to assess the condition on a single patient basis. As a result, the aim of this work consists in proposing an alternative single-subject approach, using a model-free complexity analysis based on the fractal nature of the rs-BOLD signal acquired with magnetic resonance imaging.

The rs-BOLD signal is produced by temporal fluctuations of blood oxygen in the brain. At rest, these fluctuations are caused by complex neuronal connective functions, that demand an increase in local oxygen consumption. When the deoxyhemoglobin to oxyhemoglobin ratio decreases, the local magnetic field is perturbed due to changes in magnetic properties, which cause the MR signal to increase. Previous studies by Logothetis et al. [21, 22] have shown that activation of apical dendrites in particular, is reflected in the BOLD signal. Greater spine densities in apical dendrites have been found in ASD [23], suggesting connection changes specifically in the cerebral cortex. Furthermore, a recent study has identified ASD-linked mutations in synaptic genes that affect excitatory neuron dendrite development and synapse function in the cortex [24]. These alterations in the structural organization and functional connectivity in ASD could be reflected in the rs-BOLD signal.

Studies on the brain rs-BOLD signal have shown that the signal contains spontaneous low frequency fluctuations (LFF) [25] that originate from physiological functions such as cerebral blood oxygenation and cerebral blood flow and volume as well as from instrument noise added during fMRI acquisition [26–30]. These LFF follow the inverse power law scaling in the frequency domain, which is a defined indication of fractality. Time signals are considered fractals when they are self-similar and auto-correlate across different time scales. The fractal dimension (FD) is considered a metric of signal complexity and it is a fundamental characteristic of deterministic systems where the fluctuations of the values are so complex that they mimic random behavior. Unlike random fluctuation from uncorrelated noise, fractal components in a physiological signal are produced from physiological needs. FD has been previously used as a descriptor of the neural activity based on hemodynamics and metabolic response [28, 31].

Fractal analysis of brain signals has been done in a range of pathologies that include epilepsy, Alzheimer’s disease and vascular dementia and acquired through different imaging modalities such as electroencephalography (EEG), magnetoencephalography (MEG) and single photon emission computed tomography (SPECT) [32–35]. Additionally, recent studies have done fractal analysis, specifically on brain rs-BOLD signals, obtaining insightful information about connectivity issues on Alzheimer Disease among others [36, 37].

In this study, we hypothesize that patients with ASD could show changes in the FD of the rs-BOLD signal and that these changes would reflect underlying alterations of neural structural organization and functional connectivity. The viability of this method to asses individual patients could help to overcome the heterogeneity issues and could have important treatment implications.

## Materials and methods

### Patients and normal controls

The data for this study was accessed through NITRC and the ABIDE-1 data-base [38]. Fifty-five ASD subjects (12.7 ± 2.4 y/o) participated in this study. Forty-five were diagnosed with Autistic Disorder, seven with Asperger’s Disorder, one with PDD-NOS and two with ASD of undetermined subtype. Diagnosis was established with the Autism Diagnostic Observation Schedule (ADOS) [39], the Autism Diagnostic Interview Revised (ADI-R) [40] as well as clinical consensus. Fifty-five age-matched (14.1±3.1 y/o) subjects were MRI scanned as typical controls. In order to avoid controls with borderline intelligence quotient (IQ), typical controls were required to score more than 85 on the IQ, less than 10 on the Social Communication Questionnaire [41] and less than 6 on the obsessive-compulsive sub-scale of the Spence Children’s Anxiety scale (SCAS) [42]. See Table 1 for phenotypic information.

**Table 1:** Phenotypic data.

	ASD subjects		Healthy Controls	
	Mean	STD	Mean	STD
**Age(y)**	12.7	2.4	14.1	3.1
**IQ**	103.1	17.7	105.9	10.4
**Male to Female ratio**	46:9		38:17	
**Right to Left handedness ratio**	41:6		44:8	
**ADI-R total**	41.6	8.7		
**ADOS severity**	6.5	2.2		

### Data acquisition and pre-processing

Resting state Blood Oxygenation Level Dependence (rs-BOLD) fMRI and anatomical data were acquired on a 3T GE Signa scanner using a 32-channel RF-coil (General Electric Healthcare, Milwaukee, WI). The anatomical data were acquired following a 3-plane localizer and a calibration scan designed for parallel imaging using a 3D inversion recovery-prepped T1-weighted pulse sequence (fSPGR, axial acquisition, TE/TR/flip angle = 1.8/15.63/15deg, 256x256 matrix with 1.2 mm slice thickness with 26 cm FOV). Resting state functional BOLD data was acquired in 10 min using an echo planar imaging (EPI) sequence with FOV = 22cm, image matrix = 64x64; flip angle = 90deg; echo time (TE) = 30 ms; repetition time (TR) = 2000ms (i.e, 0.5Hz temporal sampling frequency); slice thickness of 3mm; and 300 temporal points. At the beginning of every scan, 4 additional data points (dummy scans) were acquired but automatically discarded to allow the system to reach steady state. The rs-BOLD data were corrected for motion artifacts using a time series general affine registration for 12 parameters (3dWarpDrive/AFNI). Posterior ROI analysis required the rs-BOLD data to be transformed into a standard space where statistical maps of anatomically defined brain regions have been defined. Skull stripped anatomical data were aligned to the TT_N27 Tailarach standard space then warped with the rs-BOLD data using a 12-point affine transformation to obtain our final dataset. The 240 regions of interest (ROIs) defined for this study were extracted from the TT_Daemon human brain atlas [43] provided with the AFNI package [44].

### Fractal analysis

Because the rs-BOLD signal has been associated with post-synaptic potentials, which are mainly localized in grey matter as opposed to action potentials more common in the white matter; we calculated FD over a grey matter mask. Fractal dimension estimation was done by calculating a voxel-wise Hurst exponent (*H*) on the grey matter mask, following the methodology proposed by Eke et al. [30]. For self-affine processes in an n-dimensional space, the Hurst exponent is related to the fractal dimension (FD) such as *FD* + *H* = *n* + 1, where *n* = 1 for a time domain signal. Therefore, in our study FD was calculated as 2 − *H*. The rs-BOLD raw signal was initially normalized, end matched and bridge de-trended following Eke’s procedure. The data was normalized by subtracting the mean from every data point while end matching and bridge detrending was achieved by subtracting from the data the line that connects the first and the last point and multiplying the data by a parabolic window (*W*(*j*)) Eq (1).

$$\begin{array}{c} W\left(j\right)=1-(\frac{2j}{N+1}-1),j=1\to N, \end{array}$$ (1)

where *N* is the number of time points.

The series were Fourier transformed to the frequency domain and the scaling exponent (beta) of the inverse power law Eq (2) calculated.

$$\begin{array}{c} {|A\left(f\right)}^{2}|\propto cf^{-\beta }, \end{array}$$ (2)

where *A* is the amplitude of the discrete Fourier transform (DFT) at frequency *f*; *β* is the spectral index and *c* is a constant. The spectral index was calculated in a frequency range from 0.08—0.16 Hz where power-law scaling behavior was consistently observed across all voxels and subjects. A previous study [28] have suggested excluding low frequency regions below 0.02 Hz due to the presence of MRI system noise in that region [26]. The spectral index calculated from this frequency range was exclusively used for signal classification while the entire signal in the time-domain was used in the final estimation of the Hurst exponent. The goodness of fit of the spectral index for the selected range of frequency in a single subject was characterized by the (*R*^2^) value (0.72±0.21 for 3740 time series voxels).

Following the dichotomous model proposed by Mandelbrot and Van Ness [45] the signals were classified as fractional Brownian motion (fBm) for *β* > 1 and fractional Gaussian noise (fGn) for *β* < 1.

The Hurst exponent on fGn signals was calculated by using the dispersional analysis proposed by Bassingthwaighte [46], which is based on the variability of the local averages of the signal over different time windows (*τ*) Eq (3). However, a scaled window variance analysis was used to calculate *H* on the fBM signals where the series, were divided in non-overlapping windows.

$$SD\left(\tau \right)=SD\left(\tau_{0}\right){\left(\frac{\tau }{\tau_{0}}\right)}^{H}$$ (3)

Signals where *β* was near 1 were extremely difficult to classify, therefore the classification method was refined using the signal summation conversion method (SSCM) described by [30]. It is important to mention here that fractal dimension estimation based on a dispersional analysis is quite robust with respect to uncorrelated noise and does not require preprocessing [46]. For instance, uncorrelated noise generated by motion artifacts will not affect the estimation of the fractal dimension.

### Z-score analysis

A voxel-based Z-scoring methodology and the Pearson Product Moment Correlation PPMC was used in the statistical analysis. The Z-score is the number of standard deviations (*σ*) a data point is above (*Z* > 0) or below the mean (*Z* < 0). The Z-score of the voxel-wise fractal dimension was calculated as: *Z*_*FD*_ = (*x* − *μ*)/*σ*. Where x is the localized voxel rs-BOLD FD and *μ* and *σ* are the voxel mean and standard deviation of that same voxel from the control group respectively. Efficacy of the Z-score is based on the assumption of normality of the data. Therefore, the data was subjected to analyses of normality such as the Kolmogorov-Smirnov test [47], Kurtosis and Skewness assessment, where voxels that didn’t fit the model were excluded from the analysis.

Additionally, the voxels that contained data from less than 11 subjects in the control group were filtered in order to sustain a statistical power of 90% over the entire mask. This approach removed less than 3.0% of the voxels in the mean mask for normal controls and it was deemed acceptable for its use in the Z-score methodology. This study was conceived as an exploratory study rather than a hypothesis driven design, therefore the overall false positive rate, accounting for multiple comparisons was controlled by selecting only regions that showed significant decrease in FD in a significant proportion of the patients. This approach is overly conservative; however, a Bonferroni correction was deemed not appropriate as we could not establish independence of the data and smoothing of the signal across voxels could significantly affect the true nature of the fractal behavior. PPMC was used to establish correlation between ROI based Z-score values and ADI-R and ADOS questionnaires. For the purpose of this study the strength of the correlation was classified as low for 0.1 < *r* < 0.3, moderate for 0.3 < *r* < 0.5 and high, for 0.5 < *r* < 1.0.

## Results

### Fractal dimension and Z-scoring

**Fig 1:**
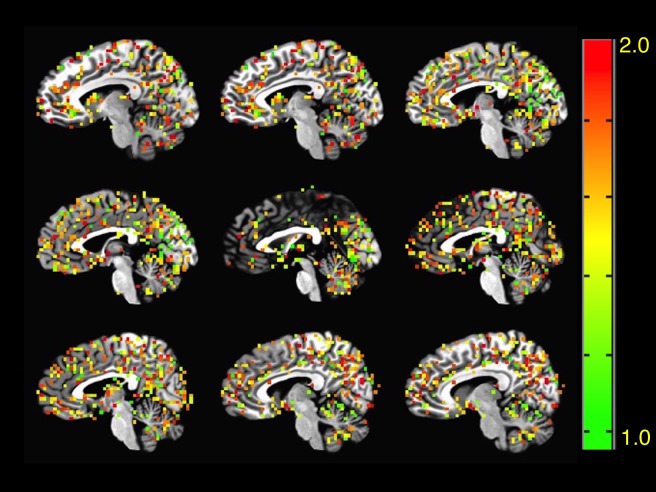
FD map of ASD. FD map over a gray matter mask of a single representative ASD patient showing regions with decreased FD and signal complexity.1 < *FD* < 2, *FD* ≠ 1.5, where *FD* = 2 − *H*. This patient in particular showed significantly reduced FD in the amygdala, the cerebellum, the thalamus and the hypothalamus among others.

**Fig 2:**
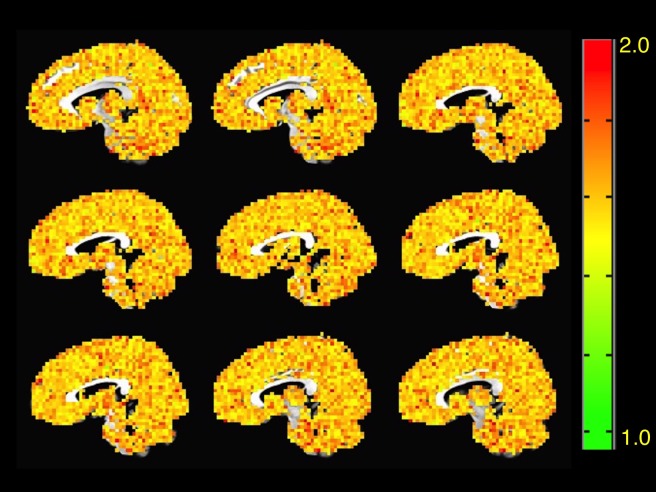
Mean FD map of healthy controls(N = 55). Mean FD map calculated from the voxel-wise mean FD value for healthy controls $\bar{FD}=1.57$, *σ* = 0.13. FD = 2-H. Grey matter mask was calculated from a probability atlas [43].

To determine regions of the brain of ASD patients with increased or decreased signal complexity respect to a typical control group, we estimated the voxel-wise FD on every subject of both groups. Following the calculation of a mean FD and standard deviation per voxel in the control group, we proceeded to calculate the Z-score of every voxel in the ASD group respect to controls. Only Z-score values greater than two were considered statistically significant for a 0.95 confidence level. Fig 1. 1 shows a montage of a typical FD-score map of a randomly selected ASD patient and Fig 2 shows the same montage for the average healthy control.

**Fig 3:**
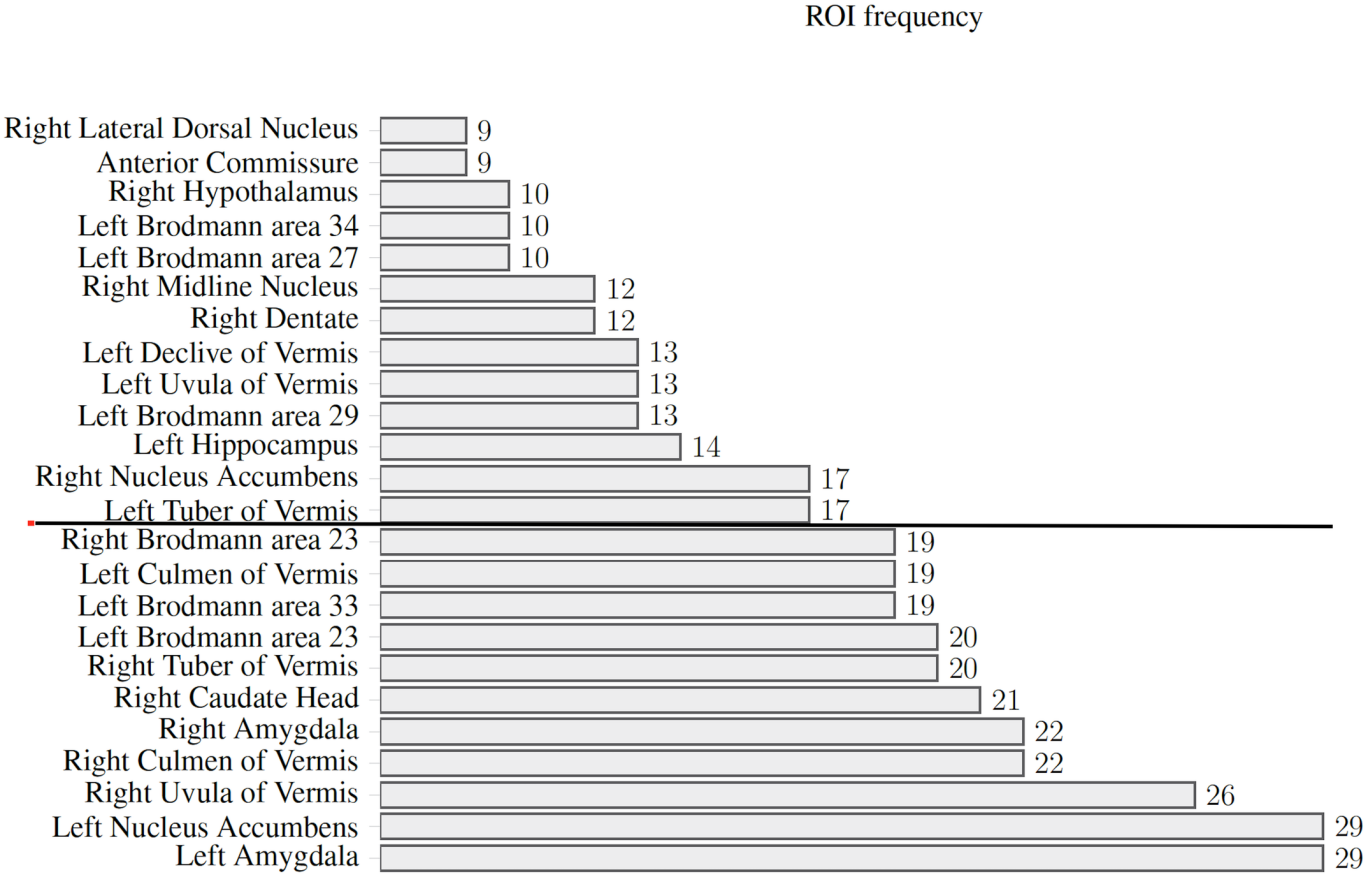
ROI frequency. Bar graph showing frequency of regions where FD decreases significantly. Statistically significant regions below the bold line are indicated.

A mean Z-score value was calculated for each of the ROIs and the ten regions the deviated the most from the control group were extracted for each patient. The frequency each region repeats among the ASD subjects is shown in Fig 3. The sample size needed to detect a difference between a Z-score value of 1 and 2 with a power of 0.90, considering a maximum standard deviation of 1.24, was 19; therefore, we only included in the analysis, regions that repeated at least in 19 patients out of the total. Table 2 shows mean Z-score, standard deviation and p-values for the ROIs that followed this criteria. Positive Z-scores were minimal in every patient and in none of the ASD subjects, we found a FD value of at least one standard deviation above the mean. This indicates that there were no regions where signal complexity significantly increased respect to controls among the ASD patients.

**Table 2:** Mean Z-score(*μ*), standard deviation(*σ*) and p-values for ROIs where FD values deviated greatest from healthy controls.

ROIs	*μ*	*σ*	p
Left Amygdala	-2.88	0.73	0.00
Left Brodmann area 23	-2.66	0.60	0.01
Left Brodmann area 33	-2.17	1.34	0.03
Left Culmen of Vermis	-2.70	1.09	0.01
Left Hippocampus	-2.32	0.85	0.02
Left Nucleus Accumbens	-2.94	1.43	0.00
Left Tuber of Vermis	-2.22	1.11	0.03
Right Amygdala	-2.72	0.83	0.01
Right Brodmann area 23	-2.64	0.57	0.01
Right Caudate Head	-2.67	0.59	0.01
Right Culmen of Vermis	-2.43	1.35	0.02
Right Nucleus Accumbens	-2.42	1.01	0.02
Right Tuber of Vermis	-2.26	1.26	0.02
Right Uvula of Vermis	-2.65	1.20	0.01

### Correlation between fractal dimension and symptom severity

To assess correlation between FD and measures of symptoms severity, we implemented a PPMC analysis. Severity of the symptoms was quantified using the scores from the ADI-R and ADOS assessment tools. Higher ADI-R and ADOS scores indicate that a patient has a greater number of items representing core deficits and greater severity of impairment. To eliminate the confounding from IQ differences between the groups, the data was IQ matched with IQ(ASD) = 103.1 ± 17.7 and IQ(controls) = 105.9 ± 10.38. Correlation coefficients were calculated between each ADI-R and ADOS scores and the normalized FD (Z_score) for 11 ROIs where FD decreased significantly in the ASD group.

The ADI-R covers the three autism domains: difficulties in verbal and non-verbal communication, impairment in social interaction and restricted and repetitive behaviors. Each domain is coded in a scale from 0 (absence of the symptom) to 3 (present in extreme form). The total combines each of the three domains and a patient is diagnosed if the three domains are above cutoff. While ADI-R is based on parental report, a trained examiner, who exposes the patient to behavioral presses that allow for the observation of behaviors associated with ASD, performs the ADOS test. Table 3 shows the correlation coefficients and p values between FD Z-score and three ADI-R measurements: ADI-R social interaction, ADI-R verbal communication and ADI-R restricted and repetitive behaviors on the selected regions of interest. Additionally, Table 4 shows the correlation coefficients and p values between FD Z-score and four ADOS measurements: ADOS social interaction, ADOS restricted and repetitive behaviors, ADOS total and ADOS severity on the selected regions of interest.

**Table 3:** Pearson correlation coefficients and p-values for ADI-R compared against regional rs-BOLD Z-score. ADI-R social interaction (ADI_SOC), ADI-R verbal communication (ADI_VERB) and ADI-R restricted and repetitive behaviors (ADI_RRB) and ADI total (ADI_TOT).

ROIs	ADI_SOC		ADI_VERB		ADI_RRB		ADI_TOT	
	r	p	r	p	r	p	r	p
Left Amygdala	0.07	0.62	0.14	0.30	-0.02	0.91	0.10	0.48
Left Brodmann area 23	0.30	0.03	0.17	0.21	-0.04	0.78	0.23	0.09
Left Brodmann area 33	0.20	0.15	-0.11	0.42	-0.10	0.50	0.04	0.80
Left Culmen of Vermis	0.11	0.42	0.02	0.88	-0.10	0.46	0.04	0.75
Left Hippocampu s	0.07	0.59	-0.04	0.76	-0.11	0.44	-0.01	0.96
Left Nucleus Accumbens	-0.02	0.87	-0.03	0.81	0.07	0.61	-0.01	0.95
Left Tuber of Vermis	0.03	0.85	0.02	0.90	-0.22	0.11	-0.04	0.79
Right Amygdala	0.25	0.07	0.31	0.02	-0.03	0.83	0.27	0.05
Right Brodmann area 23	0.13	0.36	0.15	0.28	0.12	0.38	0.17	0.22
Right Caudate Head	0.12	0.40	0.03	0.83	-0.11	0.44	0.05	0.72
Right Culmen of Vermis	0.03	0.85	0.06	0.69	-0.16	0.26	0.00	0.98
Right Nucleus Accumbens	0.09	0.50	0.15	0.28	0.24	0.09	0.18	0.19
Right Tuber of Vermis	0.07	0.61	0.06	0.66	-0.10	0.48	0.04	0.77
Right Uvula of Vermis	0.30	0.03	0.13	0.35	-0.02	0.88	0.22	0.11

**Table 4:** Pearson correlation coefficients and p-values for ADOS compared against regional rs-BOLD Z-score. ADOS social interaction (ADOS_SOC), ADOS restricted and repetitive behaviors (ADOS_RRB), ADOS total (ADOS_TOT) and ADOS severity(ADOS_SEV).

ROIs	ADOS_SOC		ADOS_RRB		ADOS_TOT		ADOS_SEV	
	r	p	r	p	r	p	r	p
Left Amygdala	0.04	0.80	0.18	0.26	0.02	0.86	0.05	0.76
Left Brodmann area 23	0.09	0.57	-0.04	0.78	0.01	0.94	0.06	0.67
Left Brodmann area 33	-0.02	0.92	-0.06	0.72	-0.07	0.63	-0.05	0.74
Left Culmen of Vermis	0.01	0.94	0.09	0.58	0.02	0.87	0.06	0.66
Left Hippocampus	-0.15	0.33	0.01	0.95	-0.09	0.52	-0.03	0.81
Left Nucleus Accumbens	-0.05	0.76	-0.07	0.64	-0.04	0.80	0.01	0.96
Left Tuber of Vermis	-0.10	0.54	-0.20	0.21	-0.15	0.29	-0.12	0.40
Right Amygdala	0.11	0.49	0.20	0.21	0.03	0.81	0.07	0.65
Right Brodmann area 23	0.17	0.29	0.16	0.31	0.12	0.43	0.15	0.31
Right Caudate Head	-0.27	0.08	-0.18	0.26	-0.19	0.19	-0.13	0.38
Right Culmen of Vermis	-0.14	0.37	-0.19	0.22	-0.17	0.25	-0.15	0.31
Right Nucleus Accumbens	-0.04	0.81	0.10	0.52	-0.07	0.65	-0.04	0.76
Right Tuber of Vermis	-0.07	0.67	0.07	0.65	-0.04	0.76	0.01	0.92
Right Uvula of Vermis	-0.08	0.60	-0.19	0.22	-0.18	0.22	-0.12	0.41

Low FD characterizes less complex signals, which has been previously associated with pathologies of the brain [37, 48–50]. We hypothesized, that as the severity of the symptoms increase, FD should decrease respect to the mean for that ROI, therefore Z-score decreases (absolute Z-score value increases) and we expect negative correlation. Positive correlation means that although FD values are all below the mean for the selected ROIs, they increase (absolute value decreases) as symptoms severity increase.

## Discussion

The present study explored the differences between ASD patients and controls in terms of signal complexity using the fractal analysis of the rs-BOLD signal. Brain connectivity is best described in a multilevel model that takes into account three distinctive levels of interaction: synaptic connections that link independent neurons, networks that connect neuronal populations and brain regions linked by fiber pathways [51]. A measure of complexity of this model constitutes an ideal indicator of multilevel and multitemporal connectivity within different brain regions [52]. Multilevel complex systems have a high degree of connectivity between its levels. The degree of connectivity between the levels is usually encoded in the system’s self-similarity. Tentatively, a healthy brain is associated with more complex signals and high FD, while a diseased or dysfunctional brain is associated with less complex signals and low FD [37, 48–50].

A recent study in our laboratory have shown regions in the brain with decreased FD in mild traumatic brain injury patients that correlates with neurological symptoms [53]. The capacity of the brain to perform real-time adaptation and processing of these connections is reflected in the local demand of glucose and oxygen consumption, which drives the brain metabolic fluctuation observed in the rs-BOLD signal.

Overall, we found reduced signal complexity in the ASD subjects with respect to controls. Out of 250 regions, 14 regions showed significantly low FD in at least 19 subjects simultaneously (see Fig 3). On average these regions had Z-scores values of −2.55 ± 0.25, or 2.55 standard deviations below the mean for that specific region. Positive Z-scores or regions where FD increased respect to controls were minimal in every patient and in none of the ASD subjects we found a FD value of at least one standard deviation above the mean. This indicates that there were no regions where signal complexity significantly increased respect to controls among the ASD patients.

In twenty-nine out of fifty ASD patients, the left amygdala and the left nucleus accumbens were among the regions with lower Z-score values, and the left nucleus accumbens reported the lowest values among all the studied ROIs. Abnormal function of the amygdala is considered a strong neurobiological marker in ASD and it is associated with deficits of social perception, affiliation and anxiety [14, 54, 55]. Our study showed decreased FD in the amygdala (*Z* − *score* = −2.8 ± 0.78) with respect to controls. However, no significant negative correlation was found between the Z-score in the amygdala and any of the metrics from the ADI-R or ADOS. Positive low correlation was detected for ADI-R social and ADOS (RRB), which means that as the severity of the symptoms increase in these areas the Z score increases and FD values or signal complexity increases. The role of the amygdala in ASD is still under scrutiny. While some authors report that diminished amygdala function correlates with deficits in social intelligence, perception and motivation, on patients with ASD [54, 56] others have reported increased amygdala activity during the perception and experience of emotion, and fear [57, 58]. It has been established that anxiety disorders occur simultaneously with with social impairment in ASD [59], creating a confounding situation in terms of determining the role of the amygdala in ASD.

The nucleus accumbens and the caudate head are functional components of the basal ganglia. These two regions showed significantly reduced fractal dimension and thus reduced signal complexity, reporting Z-scores of (*Z* − *score* = −2.68 ± ±1.22) and (*Z* − *score* = −2.67 ± 0.59) respectively. We found significant low correlation between the caudate head and ADI-RRB (r = -0.11), ADOS_Social (r = -0.27), ADOS_RRB (r = -0.18), ADOS_TOTAL (r = -0.19) and ADOS_Severity (r = -0.13), implying that signal complexity in the region decreases as symptoms severity increases. The basal ganglia is known to be involved in voluntary movement and social behavior. Previous animal studies have shown that deletion of the SAPAP3 gene in mice leads to defective neuronal communication in the basal ganglia and repetitive behaviors implicated in obsessive compulsive disorders and ASD [60]. Furthermore, structural abnormalities in the basal ganglia have been correlated to behavioral features of ASD [61] and deep brain stimulation (DBS), targeting the frontocortical-basal ganglia circuitry, have been used in the treatment of low functioning ASD patients [62]. Analysis of the fractal dimension in the basal ganglia could become a diagnostic marker to assess individual patients in order to determine whether this patient could benefit from a targeted treatment as DBS.

Several regions of the cerebellum in the ASD cohort showed significant reduced FD, specifically in the vermis (Table 2). These results mildly correlate with the ADIRRB and ADOSRRB metrics, hence, when severity of the symptoms increase in those domains, the FD and signal complexity decreases (See Tables 3 and 4). Reduced complexity in this region was expected as the cerebellum is the most consistent region of neural abnormality in autism. Postmortem studies in individuals diagnosed with autism revealed reduced number of Purkinje neurons [63, 64] while in-vivo MRI studies have found that cerebellar gray matter volume was reduced relative to normal in ASD patients. The cerebellar vermis, which is a predominately a gray matter region, is also frequently reported as atrophied [65]. Impaired cerebellar dysfunction, if detected in an early stage, could benefit from interventional approaches that can help the patients develop compensatory strategies. Analysis of the FD relative to controls could add important information in the process of deciding whether specific patients could benefit from these interventions.

We acknowledge two main limitations of this study. First, most of the ASD patients included in this study received psychotropic medication. Since the use of medication is extraordinarily high in ASD, excluding subjects under medication would potentially lead to an unrepresentative sample size. We did not explore how the use of different classes of medications affected signal complexity in our regions of interest, which could be confounding the results. Secondly, the rs-BOLD signal was acquired at a relatively low sampling frequency (0.5 Hz). Accuracy of the fractal analysis is based on the ability to capture true dynamics of the processes being studied which could be achieved with higher sampling frequencies and by increasing the sampling time. Our sampling frequency was technically limited by the EPI acquisition as it limits the number of slices that can be acquired on a single shot. To cover the human brain at a resolution of 3mm per slice we were required to read around 30 slices on a single shot, which limited the minimum TR to 1.7 s (0.58 Hz). Additionally, we took into account the RF pulses and the safety limits required for human studies. We would recommend for future studies newer acquisition techniques such as multi-band EPI as they are able to achieve full brain sampling rates up to 2.5 Hz.

## Conclusions

This study shows how the fractal dimension analysis of the fluctuations in the brain oxygen demand appears to provide additional patient-specific brain focal information that can be used to assess and possibly monitor ASD patients. Previous functional imaging approaches have focused exclusively on characterization of brain networks through group-based statistics. That approach, while providing important information of the disorder as a whole, failed to succeed in providing single patient assessment. A measure of complexity as FD could provide a method to assess brain connectivity in ASD patients. In this study, we were able to find regions in the brain with reported decreased signal complexity using the FD methodology. These regions have been previously reported as dysfunctional for ASD patients and correlated with behavioral assessments. The method we have proposed is able to provide additional information of ASD in a non-invasive and fast manner and could hopefully help in deciding whether a patient could benefit from targeted treatments and interventional techniques.
